# Obstetrician-Assessed Maternal Health at Pregnancy Predicts Offspring Future Health

**DOI:** 10.1371/journal.pone.0000666

**Published:** 2007-08-01

**Authors:** Debbie A. Lawlor, Susan Morton, G. David Batty, Sally Macintyre, Heather Clark, George Davey Smith

**Affiliations:** 1 The MRC Centre for Causal Analyses in Translational Epidemiology, Department of Social Medicine, University of Bristol, United Kingdom; 2 School of Population Health and Liggins Institute, University of Auckland, Auckland, New Zealand; 3 MRC Social and Public Health Sciences Unit, University of Glasgow, Glasgow, Scotland; 4 Department of Psychology, University of Edinburgh, Edinburgh, Scotland; 5 The Dugald Baird Centre for Research on Women's Health, University of Aberdeen, Scotland; Wayne State University School of Medicine, United States of America

## Abstract

**Background:**

We aimed to examine the association between obstetrician assessment of maternal physical health at the time of pregnancy and offspring cardiovascular disease risk.

**Methods and Principal Findings:**

We examined this association in a birth cohort of 11,106 individuals, with 245,000 person years of follow-up. We were concerned that any associations might be explained by residual confounding, particularly by family socioeconomic position. In order to explore this we used multivariable regression models in which we adjusted for a range of indicators of socioeconomic position and we explored the specificity of the association. Specificity of association was explored by examining associations with other health related outcomes. Maternal physical health was associated with cardiovascular disease: adjusted (socioeconomic position, complications of pregnancy, birthweight and childhood growth at mean age 5) hazard ratio comparing those described as having poor or very poor health at the time of pregnancy to those with good or very good health was 1.55 (95%CI: 1.05, 2.28) for coronary heart disease, 1.91 (95%CI: 0.99, 3.67) for stroke and 1.57 (95%CI: 1.13, 2.18) for either coronary heart disease or stroke. However, this association was not specific. There were strong associations for other outcomes that are known to be related to socioeconomic position (3.61 (95%CI: 1.04, 12.55) for lung cancer and 1.28 (95%CI:1.03, 1.58) for unintentional injury), but not for breast cancer (1.10 (95%CI:0.48, 2.53)).

**Conclusions and Significance:**

These findings demonstrate that a simple assessment of physical health (based on the appearance of eyes, skin, hair and teeth) of mothers at the time of pregnancy is a strong indicator of the future health risk of their offspring for common conditions that are associated with poor socioeconomic position and unhealthy behaviours. They do not support a specific biological link between maternal health across her life course and future risk of cardiovascular disease in her offspring.

## Introduction

There is a substantial body of evidence that maternal cumulative life course exposures affect her offspring's health during the antenatal and perinatal periods and in infancy. Woman's reproductive outcomes (miscarriage, gestational age, offspring birthweight, perinatal mortality and morbidity) are affected both by her socioeconomic circumstances at the time of pregnancy and the circumstances to which she was exposed to as a child [1]. Further, maternal birthweight is associated with that of her offspring across several generations irrespective of changes in socioeconomic circumstances, and it has been estimated that 12% of fetal growth restriction in the developed world is attributable to the ‘effect’ of the mother's own birthweight on that of her offspring [2]–[5]. It has been suggested that the stronger association between maternal height and offspring birthweight, than between paternal height and offspring birthweight [5], reflects an impact of accumulated environmental influences on maternal reserves, constitution and/or nutritional status during childhood growing years on her offspring [6]. Finally, maternal ill-health during her childhood and early adulthood are related to her offspring birthweight, gestational age and perinatal mortality [7], [8]. Taken together these findings demonstrate the importance of maternal childhood growth, development and health not only for her future health and vitality but also for the health of her offspring in early life [7].

There is also consistent evidence that early life factors are associated with adult chronic disease, in particular cardiovascular disease [9]. Individuals from poorer social backgrounds at birth or in childhood have greater cardiovascular disease risk, independently of their adult socioeconomic position [10], [11]. Studies in different populations have shown an inverse association between birthweight and cardiovascular disease that is independent of potential confounding factors [9], and indicators of infant and childhood environmental exposures appear to be related to future cardiovascular disease risk [9]. However, the utility in public health terms of an association between birthweight and later cardiovascular disease outcomes has been questioned on the basis that the magnitude of the associations are modest and in general birthweight is difficult to modify. There is thus increasing interest in identifying the more distal factors that influence birthweight and offspring cardiovascular health and that may be amenable to modification. Maternal physical health and well-being at the time of pregnancy may be one such exposure.

Given that maternal health, reflecting the accumulation of social, environmental and biological exposures in her life course, has an effect on her offspring' birthweight and early infant health, and these factors are predictive of subsequent cardiovascular disease risk, it is plausible that maternal health will influence her offspring's future cardiovascular health. To our knowledge no previous study has examined the association of maternal health and vitality with future offspring cardiovascular disease risk. The aim of this study is to examine the association between obstetrician reports of maternal physical health at the beginning of pregnancy and offspring cardiovascular disease in a large cohort of individuals who were born in Aberdeen, Scotland in the 1950s. Our hypothesis is that the offspring of those mothers described by obstetricians as having poor physical health will have a greater risk of cardiovascular disease. Further, we hypothesise that this association will be specific to cardiovascular disease (ie. it will not be present for other diseases). A specific association with cardiovascular disease would be supportive of our biological hypothesis linking maternal health across her life course with subtle effects on her metabolic and cardiovascular health at the time of pregnancy, which influence fetal growth and development in utero and result in greater risk of future cardiovascular disease in her offspring.

## Methods

Data from the *Aberdeen Children of the 1950s* cohort study were used. Described in detail elsewhere [12], [13], the cohort is based on participants in the Aberdeen Child Development Survey (ACDS) [14] which collected data on the parental and childhood characteristics of 14,938 children who were in Aberdeen primary schools in 1962.[14] The ACDS was representative of Aberdeen primary school children in the early 1960s. For the 12,150 of these children who were born in Aberdeen, comprehensive information was abstracted from the Aberdeen Maternity and Neonatal Databank (AMND) about the course of their mother's pregnancy and the children's physical characteristics at birth.[14] The AMND holds research level obstetric and perinatal data for all births that have occurred in Aberdeen city between 1949 and the present day (http://www.abdn.ac.uk/dugaldbairdcentre/databank/). The 12,150 individuals born in Aberdeen between 1950 and 1956, and who took part in the ACDS, form the index members of the *Aberdeen Children of the 1950s* cohort.[12], [13] In 1999 this cohort was revitalised. Study members were traced through the General Register Office (GRO) (Scotland) and 97% have been successfully traced.[12], [13] This cohort is representative of individuals born in Aberdeen between 1950–1956 and who remained resident and attended primary school in that city up to 1962.

We have used obstetrician assessments of maternal general health and physique at their first antenatal care visit as our measure of maternal general health and vitality. Senior obstetricians classified the women at their first antenatal clinic attendance into one of five categories (‘A’ denoting very good physical grade; ‘E’ denoting very poor physical grade). Owing to small numbers in the extreme categories this variable was collapsed to three groups (very good/good, average, poor/very poor physical grade). Up until 1952 this assessment was based on a detailed assessment, by the senior obstetrician, of posture, muscle development and general appearance of vitality taking into account condition of skin, eyes, hair and teeth. After 1952 the categories A–E only were recorded (i.e. without the detailed descriptions) and it is unclear on what basis these assessments were made. However, study documentation indicates that senior obstetricians were instructed to apply the same criteria that had been used prior to 1952. Our study participants were all born between 1950–1956, i.e. close to the period during which there was greater documentation of how maternal physical grade was assessed.

We examined the association of this maternal measure of physical health and vitality with maternal gravidity, height, age, marital status, complications of pregnancy and the birthweight of her offspring, all of which we would expect to be associated with maternal health and vitality. Given the possibility for change in the methods used to assess mother's physical grade during the period of data collection, we have undertaken stratified analyses (births 1950–1952 versus births 1953–1956) to determine whether the distribution of the variable and its effects on offspring cardiovascular disease outcomes varied between these two time periods.

Data on birthweight, gestational age, maternal height (nearest inch), father's occupational social class at birth, gravidity, pregnancy induced hypertension, antepartum haemorrhage and maternal age at birth were abstracted from the Aberdeen Maternal and Neonatal Database (AMND).[12] The participant's intrauterine growth rate was estimated by calculating sex and gestational age (in weeks) internally standardised z (standard deviation) scores. Height and weight (recorded in inches and pounds, respectively) at school entry (mean age 5 years) were measured and age and sex internally standardised z-scores, based on three-month age categories, were derived for height and weight.

In 1999 we began tracing study members through the General Register Office (GRO) (Scotland) and 97% have been successfully traced.[12] Traced participants have been linked to the Scottish Morbidity Register (SMR01), which provides information, including International Classification of Diseases (ICD) coded diagnoses, for all admissions to hospitals in Scotland. A recent audit has demonstrated greater than 90% accuracy for the SMR01 data.[15] We defined a participant as a case if they had a primary or secondary (i.e. co-morbidity) diagnosis of CHD or stroke. The inclusion of secondary diagnosis ensures that anyone with documented evidence of CHD or stroke is included as a case. Thirty-three (10%) of the CHD cases and 21 (19%) of the stroke cases were secondary diagnoses. Participants have also been linked to the National Health Service Central Register (NHSCR), which provides death certificate details. We defined anyone as dying from an outcome of interest if this outcome appeared as an underlying or contributory cause on the death certificate. Just three of the CHD deaths were contributory and none of the stroke deaths were (all were underlying causes). When we repeated all of the analyses either with those participants whose outcome was based on a secondary hospital diagnosis or a contributory (but not underlying) cause of death treated as non-cases or excluded from the analyses the results did not differ from those presented here. The codes used to define CHD (myocardial infarction or angina) were 410-414, 429.2 (ICD-9) and I20-25, I51.6 (ICD-10) and those used to define stroke were 430-438 (ICD-9) and I60-I69, G45 (ICD-10).

Because of the established strong association of early life socioeconomic position with later cardiovascular disease,[16] and the fact that maternal physical grade is likely to be associated with socioeconomic position we examined the specificity of the association to determine the effect of residual confounding on any association.[17] This test of specificity will provide a more robust examination of potential residual confounding than can be achieved by adjustment for available covariables in any one dataset.[17] We examined the association of maternal physical grade with unintentional injury (ICD9: E800-E929; ICD10: V01-X59) and lung cancer (ICD-9: 162, ICD-10: C34), both of which are related to adverse socioeconomic position [18], [19] and finally with breast cancer (ICD-9: 174, ICD-10: C50), which is not related to socioeconomic position.

### Statistical methods

Cox proportional hazards regression models were used, with participants' age as the time axis. Since the SMR01 records of hospital admissions only begin in 1981, the follow-up period began on 1^st^ January 1981. Participants were omitted from the analyses if they died (N = 116), emigrated to anywhere outside Scotland (N = 927) or experienced a non-fatal stroke or CHD (N = 1) prior to 1^st^ January 1981–our start of follow-up, when the hospital admissions data became reliable in Scotland. The distribution of maternal physical grade did not differ by those who were excluded or included (p = 0.2), nor did the distributions of complications of pregnancy, maternal age at birth, gestational age or birth weight (all p-values>0.2). However, offspring who were excluded because of early death or migration were slightly less likely to be from manual social classes at birth (70% versus 79%, p<0.001). With these exclusions 11,106 (91%) of the original cohort remained in the analysis.

Contributions to risk were censored at the earlier of: (i) first episode of the outcome of interest (if an individual had repeated hospital admissions or a fatal event following an earlier admission they were censored at the first event); (ii) emigration date (this includes emigration to England or Wales); (iii) death from a cause other than the outcome of interest; (iv) 31 December 2003. Hospital admissions occurring in England and Wales cannot be obtained, which means that individuals who migrated to England and Wales are considered in the main survival analyses to be no longer at risk from the date that they move. For the emigration date of those moving to England or Wales we used their first posting date (the date that they first appear on health authority lists as being registered with a general practitioner) with a general practitioner from England or Wales. In all analyses we used robust standard errors, taking account of possible clustering within siblings (including twins or higher order multiple births), to calculate p-values and 95% confidence intervals.

### Management of missing data

We used multiple multivariate imputation, using all other covariables, the log of survival time and the censoring indicator, to impute values for those variables with some missing data (maternal physical grade 25% missing; gestational age 10% missing, fathers occupational social class 5% missing, childhood anthropometric measurements 3% missing–see table 1).[20] We used switching regression in Stata as described by Royston,[20] and carried out 20 cycles of regression switching and generated 20 imputation datasets. This approach creates a number of copies of the data (in this case we generated 20 copies) each of which has values that are missing imputed with an appropriate level of randomness using chained equations.[20] The results are obtained by averaging across the results from each of these datasets using Rubin's rules and the procedure takes account of uncertainty in the imputation as well as uncertainty due to random variation (as undertaken in all multivariable analyses).[20] This method assumes that data are either ‘missing completely at random’ or are ‘missing at random’, but are not ‘missing not at random’ (i.e. it assumes that the probability of missing data does not depend on the outcome of interest). Although this is never possible to test this assumption, in this particular case it seems unlikely that the probability that data on maternal physical grade, gestational age, father's occupation and the individual's size when they were aged 5 are dependent on their later risk of cardiovascular disease, once other exposures are taken into account, since risk of cardiovascular disease would not have been apparent when these measures were taken and we have near complete follow-up. We also undertook two further sets of analyses: (i) all analyses were repeated on the complete dataset sub-sample (N = 7060); (ii) analyses were repeated on complete dataset sub-sample and we undertook weighted analyses using inverse probability (of having missing outcome data) weights in all regression models.[21] Results for both of these sets of analyses were less precise than those combining the series of datasets with some multivariate imputed data, but the point estimates were essentially the same. In this paper for all of the descriptive statistics (tables 1–2) only those with complete data are in included, of inferential analyses (tables 3–5) the results are those obtained using the multivariate multiple imputation methods. All analyses were conducted using Stata version 9.2.

**Table 1:** Maternal and early life characteristics of cohort participants. N=11,106 [except where there is missing data as indicated^a^]

		Females N = 5411	Males N = 5695
Maternal physical grade N (%)^a^	Very good or good	2171 (53.7)	2320 (54.3)
	Mediocre	1483 (36.7)	1577 (36.9)
	Very bad or bad	386 (9.6)	375 (8.8)
Social class at birth N (%)^a^	I&II (highest)	480 (9.4)	524 (9.7)
	III NM	581 (11.4)	626 (11.6)
	III M	2409 (47.3)	2474 (45.7)
	IV	740 (14.5)	827 (15.3)
	V (lowest)	881 (17.3)	959 (17.7)
Gravidity N (%)	1	1777 (32.8)	1862 (32.7)
	2	1544 (28.5)	1649 (29.0)
	3	1000 (18.5)	1014 (17.8)
	4	551 (10.2)	555 (9.8)
	> = 5	539 (10.0)	615 (10.8)
Birth outside marriage N (%)	No	5154 (95.2)	5453 (95.8)
	Yes	257 (4.8)	242 (4.2)
Maternal age at birth (years) N (%)	15–19	245 (4.5)	262 (4.6)
	20–24	1707 (31.6)	1763 (31.0)
	25–29	1701 (31.4)	1763 (31.0)
	30–34	1114 (20.6)	1226 (21.5)
	35–39	484 (8.9)	512 (9.0)
	> = 40	160 (3.0)	169 (3.0)
Maternal Height (inch) N (%)	< = 60	1399 (25.9)	1481 (26.0)
	61	850 (15.7)	906 (15.9)
	62	977 (18.0)	1009 (17.7)
	63	769 (14.2)	837 (14.7)
	64	670 (12.4)	680 (11.9)
	> = 65	746 (13.8)	782 (13.7)
Pregnancy induced hypertension N (%)	No	4492 (83.0)	4731 (83.1)
	Yes	919 (17.0)	964 (16.9)
Antepartum haemorrhage N (%)	No	5298 (97.9)	5555 (97.5)
	Yes	113 (2.1)	140 (2.5)
Gestational age (weeks) N (%)^a^	<37	323 (6.7)	365 (7.1)
	37–40	3447 (71.1)	3679 (71.9)
	>40	1077 (22.2)	1070 (20.9)
Multiple birth	Yes	146 (2.7)	139 (2.4)
Birth weight (kg)	Mean (SD)	3.23 (0.50)	3.36 (0.51)
Childhood height (M)^a^	Mean (SD)	1.08 (0.10)	1.09 (0.11)
Childhood weight (Kg)^a^	Mean (SD)	19.55 (9.83)	20.03 (9.71)
Childhood BMI (Kg/m^2^)^a^	Mean (SD)	16.30 (1.97)	16.55 (1.81)

a For these variables there is missing data as follows:

Social class at birth 606 (320 female&285 male) 5% with missing data

Maternal physical grade 2795 (1371 female&1423 male) 25% with missing data

Gestational age 1145 (564 female&581 male) 10% with missing data

Birth weight 19 (8 female&11 male) 0.2% missing data

Childhood height, weight and body mass index 357 (157 female and 200 male) 3.2% with missing data

**Table 2:** Distribution of obstetrician's report of maternal physical grade by birth year of offspring. N=8312 [analyses only conducted on those with complete data]

	Maternal physical grade N (%)	
	Offspring born 1950–1952 N = 3455	Offspring born 1953–1956 N = 4857
Very good/good	1778 (51.5)	2713 (55.9)
Mediocre	1324 (38.3)	1736 (35.7)
Bad/very bad	353 (10.2)	408 (8.4)
χ^2^_2 d.f_ = 18.1 p<0.001		

**Table 3:** Maternal and offspring characteristics by maternal physical grade at the time of pregnancy. N=11,106 [with imputations for missing data in maternal physical grade (25%), fathers occupational social class (5%), birthweight z score (10%), childhood weight and height z-scores (3.2%)]

	N (%) or mean (SD) by maternal physical grade			P trend
	Very good or good N = 5919	Mediocre N = 3943	Bad or very bad N = 1244	
**Dichotomous variables N (%)**				
Manual social class	4368 (73.8)	3399 (86.2)	1157 (93.0)	<0.001
Gravidity > = 4	479 (8.1)	639 (16.2)	373 (30.0)	<0.001
Maternal age <20 years	385 (6.5)	197 (5.0)	51 (4.1)	0.001
Maternal age >34 years	379 (6.4)	438 (11.1)	174 (14.0)	<0.001
Pregnancy induced hypertension	1296 (21.9)	733 (18.6)	170 (13.7)	<0.001
Antepartum haemorrhage	118 (2.0)	83 (2.1)	30 (2.4)	0.21
Prematurity (gestational age <37 weeks)	331 (5.6)	276 (7.0)	141 (11.3)	<0.001
Born outside of marriage	207 (3.5)	201 (5.1)	116 (9.3)	<0.001
Multiple birth	143 (2.4)	100 (2.5)	42 (3.3)	0.20
**Continuous variables mean (SD)**				
Maternal height (inch)	62.4 (2.2)	61.4 (2.2)	60.6 (2.3)	<0.001
Birthweight z-score	0.03 (0.97)	−0.09 (0.99)	−0.23 (1.00)	<0.001
Childhood height z-score	0.15 (1.03)	−0.09 (0.94)	−0.33 (1.12)	<0.001
Childhood weight z-score	0.10 (1.22)	−0.07 (0.80)	−0.14 (1.22)	<0.001

**Table 4 pone-0000666-t004:** Associations of maternal physical grade at the time of pregnancy with CHD and stroke risk in offspring. N=11,106 [with imputations for missing data in maternal physical grade (25%), fathers occupational social class (5%), birthweight z score (10%), childhood weight and height z-scores (3.2%)]

Maternal physical grade at pregnancy	N cases	Hazard ratio for cardiovascular disease (95% CI)					
		Model 1	Model 2	Model 3	Model 4	Model 5	Model 6
**CHD**							
Very good/good	133	1	1	1	1	1	1
Mediocre	121	1.28 (0.96, 1.71)	1.22 (0.92, 1.63)	1.22 (0.92, 1.63)	1.22 (0.92, 1.63)	1.20 (0.90, 1.61)	1.17 (0.88, 1.56)
Bad/very bad	48	1.95 (1.33, 2.87)	1.63 (1.11, 2.40)	1.63 (1.11, 2.40)	1.63 (1.10, 2.40)	1.56 (1.06, 2.29)	1.55 (1.05, 2.28)
P trend	302	<0.001	<0.001	<0.001	<0.001	0.003	0.003
**Stroke**							
Very good/good	49	1	1	1	1	1	1
Mediocre	43	1.34 (0.82, 2.20)	1.22 (0.75, 2.01)	1.21 (0.74, 2.01)	1.22 (0.75, 2.01)	1.21 (0.74, 2.00)	1.19 (0.73, 1.95)
Bad/very bad	17	2.18 (1.13, 4.19)	2.03 (1.05, 3.93)	2.03 (1.05, 3.93)	2.04 (1.05, 3.94)	1.97 (1.02, 3.79)	1.91 (0.99, 3.67)
P trend	109	0.001	0.04	0.04	0.04	0.05	0.08
**CHD or stroke**							
Very good/good	174	1	1	1	1	1	1
Mediocre	159	1.32 (1.02, 1.71)	1.19 (0.92, 1.55)	1.19 (0.92, 1.55)	1.19 (0.92, 1.55)	1.19 (0.92, 1.55)	1.16 (0.89, 1.51)
Bad/very bad	64	2.10 (1.51, 2.92)	1.69 (1.23, 2.35)	1.68 (1.22, 2.35)	1.69 (1.23, 2.35)	1.62 (1.17, 2.25)	1.57 (1.13, 2.18)
P trend	397	<0.001	<0.001	<0.001	<0.001	<0.001	<0.001

Model 1: Adjusted for year of birth and sex

Model 2: Adjusted for year of birth sex and offspring birthweight z-score only

Model 3: As model 2 plus indicators of socioeconomic position (father's occupational social class, gravidity, born outside of marriage, maternal age)

Model 4: As model 3 plus maternal height

Model 5: As model 4 plus complications of pregnancy (pregnancy induced hypertension, antepartum haemorrhage)

Model 6: As model 5 plus childhood (mean age 5) weight and height

**Table 5:** Test of specificity of association with cardiovascular disease outcomes: associations of maternal physical grade at the time of pregnancy with unintentional injury, lung cancer and breast cancer in offspring. N=11,106 (N=5,412 women for association with breast cancer) [with imputations for missing data in maternal physical grade (25%), fathers occupational social class (5%), birthweight z score (10%), childhood weight and height z-scores (3.2%)]

	Hazard ratio (95% confidence interval) for different outcomes	
	Model 1	Model 2
**Unintentional injury N = 1043**		
Very good/good	1	1
Mediocre	1.21 (1.04, 1.41)	1.14 (0.98, 1.32)
Bad/very bad	1.41 (1.14, 1.74)	1.28 (1.03, 1.58)
P trend	<0.001	0.02
**Lung cancer N = 28**		
Very good/good	1	1
Mediocre	1.92 (0.67, 5.23)	1.48 (0.49, 4.51)
Bad/very bad	4.611.40, 15.10)	3.61 (1.04, 12.55)
P trend	0.01	0.05
**Breast cancer N = 76, in 5421 women**		
Very good/good	1	1
Mediocre	1.09 (0.67, 1.79)	1.10 (0.67, 1.84)
Bad/very bad	1.02 (0.46, 2.31)	1.10 (0.48, 2.53)
P trend	0.82	0.72

Model 1: Adjusted for sex for unintentional injury and lung cancer and unadjusted for breast cancer

Model 2: Adjusted for sex, birthweight, father's occupational social class, gravidity, born outside of marriage, maternal age, maternal height, pregnancy induced hypertension, antepartum haemorrhage, childhood height and weight

### Ethics

The Scottish multi-centre research ethics committee and local research ethics committees plus the Scottish Privacy Advisory Committee approved the revitalisation of the *Children of the 1950s cohort.* All record linkage was undertaken by ISD, who provided us with an anonymised dataset for analysis.

## Results

**Table 1** shows the maternal and early life characteristics of cohort members. A greater proportion of women whose offspring were born between 1950–1952 (period with a definite physical examination) were defined as having poor/very poor physical grade than those whose offspring were born 1953–1956 (**Table 2**). While these differences reached statistical significance absolute differences were small.

**Table 3** shows the association of maternal physical grade with maternal and offspring perinatal characteristics. There were strong linear associations across the three categories of maternal physical grade for most characteristics. The only exceptions were antepartum haemorrhage and multiple birth. Mothers who experienced an antepartum haemorrhage and those with a multiple birth were somewhat more likely to be graded as having poor physical grade. However, neither of these associations reached conventional levels of statistical significance. Women who were rated by their obstetrician as having poor/very poor physical grade were more likely to have husbands in manual social classes, more likely to be gravida 4 or more, to be of older age and to have experienced pregnancy induced hypertension than women whose physical grade was rated as good/very good. These women also had a greater risk of delivering a preterm infant, of having a lower birthweight infant and a child with shorter stature and lower weight at the time of school entry. Those rated as average grade were intermediate on these characteristics. None of these associations varied between those women whose offspring were born between 1950–1952 and those whose offspring were born between 1953–1956 (all p-values for interaction >0.5).

At the start of the follow-up period (1981) there were 11,106 members of the cohort alive and believed to be resident in Scotland. Over the follow-up period they contributed 245,000 person years of risk. Among these participants there were 302 (53 fatal) cases of CHD, 109 (4 fatal) cases of stroke and 397 (57 fatal) cases of either a CHD or stroke (14 women experienced both a CHD and stroke event during the follow-up period and thus the combined outcome has fewer events than the sum of CHD and stroke events; these women were censored at the date of their first event irrespective of whether this was CHD or stroke in the combined analyses).

The associations of maternal physical grade with both CHD and stroke were the same in both women and men (stratified analyses were essentially the same in both gender but less precisely estimated that for analyses with both sexes combined, particularly for women; all p-values for interaction with gender were >0.4). We therefore pooled data for both women and men in all further analyses. The association between maternal physical grade and offspring cardiovascular disease did not differ by whether the offspring was born between 1950–1952 or between 1953–1956 (p-values for interaction with period of birth >0.4), therefore in multivariable analyses all data, irrespective of year of birth, are combined. Adjustment for year of birth is made in all analyses.

**Table 4** shows the association between maternal physical grade and offspring risk of CHD, stroke and both combined with adjustment for potential covariables. In year of birth and sex adjusted models there were strong associations between maternal physical grade and risk of offspring CHD, stroke and both combined. These associations attenuated with adjustment for all potential confounders, but positive associations remained. Maternal height at the time of pregnancy was inversely associated with offspring cardiovascular disease in sex adjusted models (hazard ratio for CHD and Stroke combined per 1 inch maternal height 0.93 [95%CI: 0.88, 0.99]), but this association attenuated with adjustment for indicators of socioeconomic position (0.97 [95%CI: 0.91, 1.03]). Additional adjustment for maternal physical grade at the time of pregnancy effectively eliminated the association of maternal height at birth with offspring risk of CHD and stroke: 0.99 [95% CI: 0.93, 1.05].

The association of maternal physical grade with cardiovascular disease outcomes was not specific. There were strong positive associations with exposures that are related to adverse socioeconomic position, but not with breast cancer, which is not associated with socioeconomic position (**Table 5**). All of the results from the multivariable analyses presented in table 4 and 5 were unchanged when they were repeated with multiple pregnancies removed.

## Discussion

We have found that a brief assessment by an obstetrician of a pregnant woman's health and vitality at the time of pregnancy is related to future cardiovascular disease risk in her offspring. Thus, the offspring of women described as having poor/very poor physical grade have increased risk of CHD and stroke compared to the offspring of women described as having better health and vitality. The association remained after adjustment for a range of potential confounding factors, including several indicators of socioeconomic position (fathers occupational class, gravidity, maternal height). However, the association was not specific since strong associations were also found for other outcomes–unintentional injury and lung cancer–that are known to be more common amongst those from adverse socioeconomic backgrounds, but not with breast cancer, which is not associated with socioeconomic position. This lack of specificity suggests that any remaining association with cardiovascular disease outcomes after adjustment for available confounders is most likely explained by residual confounding related to socioeconomic position.[17]

### Study strengths and limitations

The main strength of this study is its large size, intergenerational data and the availability of adult disease outcomes in offspring. We have used a measure of maternal health and vitality that has not been validated. However, the obstetricians grading was based on a series of relevant physical examination findings (including posture, muscle development, eyes, skin tone and hair) for those cohort members born between 1950–1952. Although the distribution of maternal grade varied a little between this group and those born later the effects of maternal physical health and physique on cardiovascular disease and other outcomes did not vary between these two groups. Further, this measure of maternal physical health and vitality related to other maternal characteristics in expected directions (**Table 3**). The results are from a single city in Scotland, with obstetricians making the observations on women who delivered infants between 1950–1956, and we cannot necessarily assume that the same findings would be obtained from obstetricians making similar assessments of physical grade in different countries or at different periods of time. However, these assessments will have been undertaken by a number of different obstetricians, but it would be interesting to see if they were replicated in other studies. 25% of the participants had missing data on our main exposure but analyses using multivariable imputation did not differ from those based on the complete data subset, suggesting that missing data has not importantly biased our findings.

### Implications of our findings

Several mechanisms might link maternal physical health and vitality to offspring cardiovascular disease risk. First, an association might be mediated via the effect of maternal health and physique on intrauterine environment and early infant health, which are associated with cardiovascular disease.[9] Second, genetic factors may predispose both mother and her offspring to cardiovascular disease. Third, the association may be an expression of the known association between childhood socioeconomic position and adult cardiovascular disease risk.[10], [16] Though the association remained despite adjustment for a range of characteristics that would directly (fathers occupational social class) or indirectly (parity, maternal height, maternal age at birth) reflect socioeconomic position the strong associations with other outcomes that are known to be influenced by socioeconomic position suggests that this may, at least in part, explain the association. Finally, mothers rated by their obstetricians as having poor physical grade are perhaps more likely to engage in health damaging behaviours and these behaviours may then be adopted by their offspring. For example, maternal smoking during pregnancy and later in the child's life is associated with increased risk of offspring smoking.[22], [23] Thus, mothers who smoked are likely to have had poorer physical appearance and their offspring's cardiovascular disease risk may be increased by their increased likelihood of becoming a smoker. An important limitation of our study is that we do not have information on maternal smoking or other behaviours in this cohort to explore whether these do indeed explain the associations we have found. We do have participant self-reported data on smoking, alcohol, weight and height collected in 2001, but for most of the cardiovascular events these occurred prior to this date and therefore we cannot explore whether these mediate any association between maternal physical grade at the time of pregnancy and later cardiovascular disease outcomes in her offspring. However, the association of maternal physical grade with lung-cancer (strongly associated with smoking) and unintentional injury (likely to be non-specifically associated with smoking because of its association with socioeconomic position) support this mechanism as at least partly responsible for the association.

To conclude our findings suggest that a simple physical assessment of mothers general health and vitality (base on skin, eyes, hair and teeth), by their obstetricians in early pregnancy is a powerful predictor of common diseases in the adult offspring that are related to adverse socioeconomic position and unhealthy behaviours such as smoking. These associations are unlikely to be biological in nature but more likely to be driven by shared adverse environments across generations. None the less they indicate the potential of antenatal assessments to identify families most at risk of future ill-health and for whom targeted health promotion might be particularly beneficial. Our findings also illustrate the value of examining specificity of associations to explore the potential of residual confounding.
